# Flagellar region 3b supports strong expression of integrated DNA and the highest chromosomal integration efficiency of the *Escherichia coli* flagellar regions

**DOI:** 10.1111/1751-7915.12296

**Published:** 2015-06-15

**Authors:** Mario Juhas, James W Ajioka

**Affiliations:** Department of Pathology, University of CambridgeTennis Court Road, CB2 1QP, Cambridge, UK

## Abstract

The Gram-negative bacterium *Escherichia coli* is routinely used as the chassis for a variety of biotechnology and synthetic biology applications. Identification and analysis of reliable chromosomal integration and expression target loci is crucial for *E. coli* engineering. Chromosomal loci differ significantly in their ability to support integration and expression of the integrated genetic circuits. In this study, we investigate *E. coli* K12 MG1655 flagellar regions 2 and 3b. Integration of the genetic circuit into seven and nine highly conserved genes of the flagellar regions 2 (*motA*, *motB*, *flhD*, *flhE*, *cheW*, *cheY* and *cheZ*) and 3b (*fliE*, *F*, *G*, *J*, *K*, *L*, *M*, *P*, *R*), respectively, showed significant variation in their ability to support chromosomal integration and expression of the integrated genetic circuit. While not reducing the growth of the engineered strains, the integrations into all 16 target sites led to the loss of motility. In addition to high expression, the flagellar region 3b supports the highest efficiency of integration of all *E. coli* K12 MG1655 flagellar regions and is therefore potentially the most suitable for the integration of synthetic genetic circuits.

## Introduction

The Gram-negative model bacterium *Escherichia coli* is capable of thriving in a wide variety of environments (Juhas *et al*., 2014a). Easily amenable to genetic manipulations, *E. coli* strain K-12 is among the most frequently used hosts for cloning and the intermediate and the final destination chassis for engineering large DNA fragments. *Escherichia coli* K-12 is also important for a number of industrial applications, biomanufacturing and metabolic engineering (Ajikumar *et al*., 2010; Zhang *et al*., 2010; Clomburg and Gonzalez, 2011; Yim *et al*., 2011; Zhou *et al*., 2012). With the advent of synthetic biology, *E. coli* K-12 has become one of the most frequently used synthetic biology host organisms (Juhas *et al*., 2013; 2014a; Juhas, 2015).

Introduction of the synthetic DNA fragments into the *E. coli* genome by chromosomal integration has many advantages over the plasmid-borne transformation (Cunningham *et al*., 2009; Marcellin *et al*., 2010). Furthermore, integration into the chromosome could be exploited for heterologous protein expression, particularly for expression of toxic proteins in *E. coli*. Work on plasmids has shown that regulation of expression is tighter when the copy number is low (Anthony *et al*., 2004; Guan *et al*., 2013). The frequently used methods of the *E. coli* chromosomal integration include the integrase-mediated recombination between the phage attachment sites (*att*) (St-Pierre *et al*., 2013) and the λ bacteriophage Red recombinase-mediated recombination employing knock-in/knock-out (KIKO) vectors (Sabri *et al*., 2013), plasmid pSB1K3(FRTK) (Juhas *et al*., 2014b) and the yeast mitochondrial homing endonuclease I-SceI (Ublinskaya *et al*., 2012). Chromosomal integration target sites differ significantly in their ability to support integration and expression of the integrated genetic circuits (Juhas *et al*., 2014b). As the traditionally used *att* sites are missing in a number of industrially important *E. coli* strains, identification and validation of the reliable chromosomal integration target sites is crucial for *E. coli* engineering. Ideally, integration target sites should be well-characterized, non-essential, conserved and highly expressed (Fraser *et al*., 1999; Baba *et al*., 2006; Vora *et al*., 2009; Kahramanoglou *et al*., 2011; Juhas *et al*., 2014b). Genes encoding flagellar functions meet all these prerequisites (Juhas *et al*., 2014b). Previous analyses of the *E. coli* K12 MG1655 flagellar regions 3a and 1 led to the identification of only three potential integration target sites (Juhas *et al*., 2014b; Juhas and Ajioka, 2015). The identification and validation of alternative integration sites is crucial for the development of a robust synthetic biology toolkit (Juhas and Ajioka, 2015). This is critical particularly for applications that require integrations of multiple genetic circuits into the chromosome. Here, we investigate the *E. coli* K12 MG1655 flagellar regions 2 and 3b. Analysis of the seven and nine highly conserved genes of the flagellar regions 2 and 3b, respectively, revealed significant variability in their suitability for integration and expression of genetic circuits. Furthermore, we show that in addition to high expression, the *E. coli* K12 MG1655 flagellar region 3b supports highest efficiency of chromosomal integration of all *E. coli* flagellar regions.

## Results and discussion

### Integration target loci in the *E. coli* flagellar regions 2 and 3b

Identification of the reliable chromosomal integration target loci is crucial for engineering *E. coli* cells (Sabri *et al*., 2013; Juhas *et al*., 2014b). Chromosomal integration target sites should be well-characterized, conserved, non-essential and highly expressed (Fraser *et al*., 1999; Baba *et al*., 2006; Vora *et al*., 2009; Kahramanoglou *et al*., 2011; Juhas *et al*., 2014b; Juhas, 2015). Genes encoding flagellar functions are considered to be among the best targets for integration of genetic circuits into the *E. coli* chromosome (Juhas *et al*., 2014b). Previous studies investigating *E. coli* K12 MG1655 flagellar regions 3a (Juhas *et al*., 2014b) and 1 (Juhas and Ajioka, 2015) led to the identification of three putative chromosomal integration target sites. Identification and validation of the alternative loci is important particularly for those biotechnology and synthetic biology applications that require integrations of multiple genetic circuits into *E. coli* chromosome.

**Fig 1:**
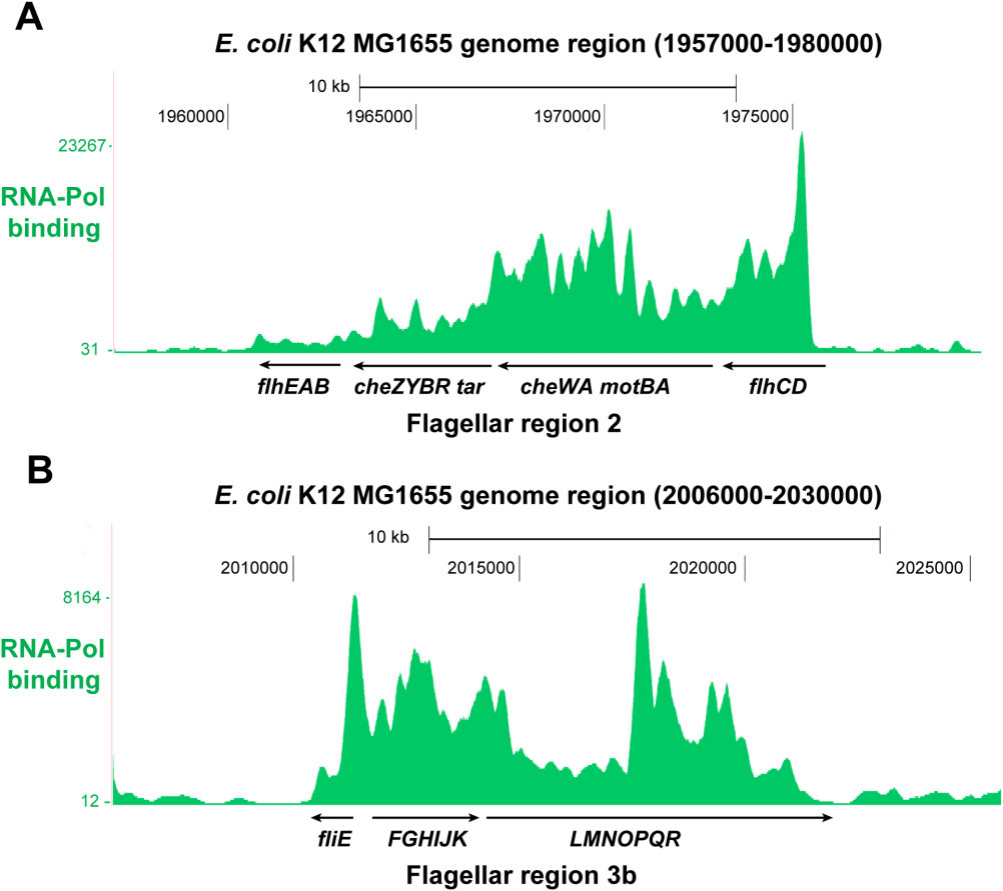
RNA polymerase binding to *E. coli* flagellar regions 2 and 3b. Figure depicts the probability of the RNA polymerase (RNA-Pol) binding (green peaks) to the *E. coli* K12 MG1655 genome regions 1957000-1980000 (A) and 2006000-2030000 (B). The investigated *E. coli* K12 MG1655 flagellar regions 2 (1962580-1978197) and 3b (2011038-2021702) show high probability of being occupied by RNA polymerase. Figure was created by uploading the ChIP-seq RNA-Pol data (Kahramanoglou *et al*., 2011) to the UCSC genome browser for *E. coli* K12 MG1655.

**Fig 2:**
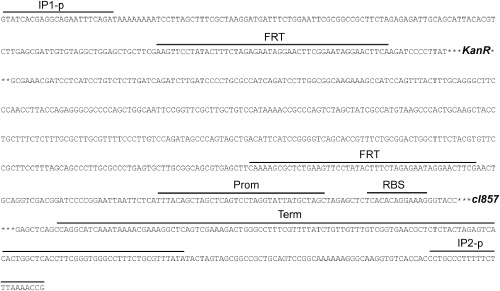
Genetic circuit Repr-ts-1. Figure shows sequence of the genetic circuit Repr-ts-1 integrated into the chromosome. IP1-p and IP2-p (integration primer parts); FRT (Flp recombinase target site); *KanR* (kanamycin resistance gene); Prom (promoter); RBS (ribosome binding site); *cI857* (temperature-sensitive lambda repressor); Term (terminator).

**Fig 3:**
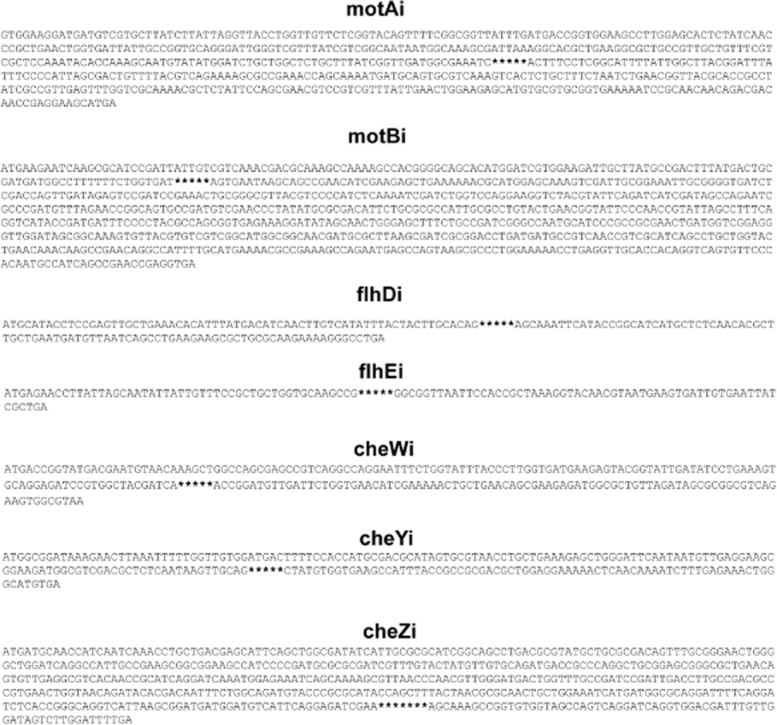
*Escherichia coli* flagellar region 2 integrations. Figure shows the sequences of the integration target sites in the *E. coli* K12 MG1655 flagellar region 2 (*motA* (motAi), *motB* (motBi), *flhD* (flhDi), *flhE* (flhEi), *cheW* (cheWi), *cheY* (cheYi). The exact positions within the target genes where the integrations occurred are highlighted with stars.

Here, we investigate *E. coli* K12 MG1655 flagellar regions 2 and 3b. *Escherichia coli* K12 MG1655 flagellar regions 2 (Fig. 1A) and 3b (Fig. 1B) show high probability of the RNA polymerase binding. This suggests that genetic circuits integrated into these regions will be strongly transcribed. *Escherichia coli* K12 MG1655 flagellar regions 2 and 3b harbour 28 open reading frames (*flhA*, *flhB*, *flhC*, *flhD*, *flhE*, *motA*, *motB*, *cheA*, *cheB*, *cheR*, *cheW*, *cheY*, *cheZ*, *tar*, *fliE*, *fliF*, *fliG*, *fliH*, *fliI*, *fliJ*, *fliK*, *fliL*, *fliM, fliN*, *fliO*, *fliP*, *fliQ*, *fliR*) (Fig. 1). Our investigation revealed that seven and five genes of the *E. coli* K12 MG1655 flagellar regions 2 and 3b, respectively, are not fitting for integration of genetic circuits because of low conservation or lack of suitable integration target sequences. The other seven genes of the flagellar region 2 (*motA*, *motB*, *flhD*, *flhE*, *cheW*, *cheY*, *cheZ*) (Table 1) and nine genes of the flagellar region 3b (*fliE*, *fliF*, *fliG*, *fliJ*, *fliK*, *fliL*, *fliM*, *fliP*, *fliR*) (Table 2) are highly conserved among *E. coli* strains, including industrially relevant strains, such as BL21-DE3, W3110, DH10B and MG1655. The function and location of the analysed chromosomal integration target loci in the *E. coli* K12 MG1655 flagellar regions 2 and 3b are shown in Fig. 1 and Tables 1 and 2. We have integrated the genetic circuit Repr-ts-1 (Fig. 2) harbouring thermosensitive lambda repressor into these loci using the modified lambda Red recombinase integration method (Juhas *et al*., 2014b).

**Table 1:** Integration target loci in the *E. coli* flagellar region 2

Gene	Function	References
*motA*	Flagellar motor component	Mohawk *et al*., 2014; Takahashi and Ito, 2014
*motB*	Flagellar motor component	Reboul *et al*., 2011; Takahashi *et al*., 2014
*flhD*	Master regulator of flagellar genes	Chatterjee *et al*., 2009; Mitra *et al*., 2013
*flhE*	Proton influx regulator via T3SS	Lee and Harshey, 2012
*cheW*	Chemotaxis signal transduction	Cashman *et al*., 2013
*cheY*	Chemotaxis response regulator, clockwise flagellar rotation	Fraiberg *et al*., 2015
*cheZ*	Phosphatase, *cheY* dephosphorylation	Freeman *et al*., 2011

**Table 2:** Integration target loci in the *E. coli* flagellar region 3b

Gene	Function	References
*fliE*	Flagellar basal body component	Dyszel *et al*., 2010
*fliF*	Membrane and supramembrane (MS)-ring collar protein, flagellar basal body	Ogawa *et al*., 2015
*fliG*	Flagellar motor switching	Lam *et al*., 2012
*fliJ*	Flagellar protein export apparatus, rotor like function	Kishikawa *et al*., 2013
*fliK*	Flagellar hook-length control	Aizawa, 2012
*fliL*	Flagellar motor output control	Partridge *et al*., 2015
*fliM*	Flagellar motor energizing	Delalez *et al*., 2014
*fliP*	Flagellar export apparatus	Boyd and Gober, 2001
*fliR*	Flagellar export apparatus	Minamino and Macnab, 1999

### High efficiency integration into *E. coli* flagellar region 3b

**Fig 4:**
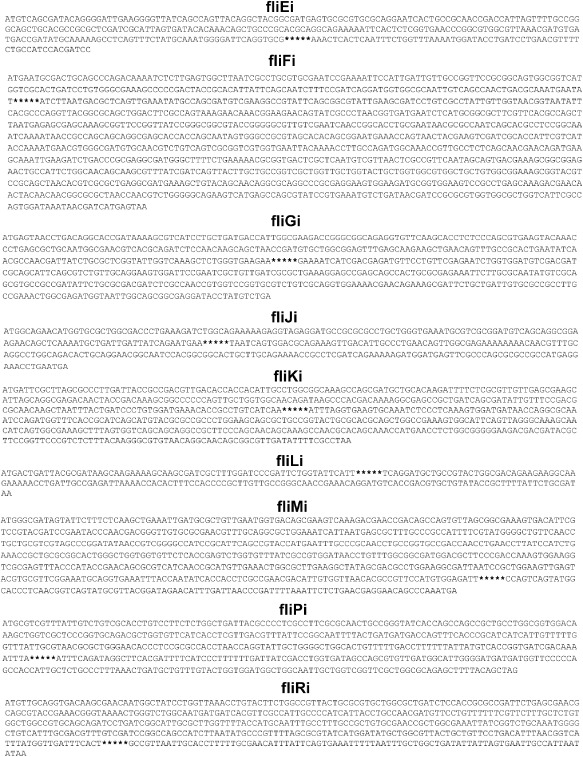
*Escherichia coli* flagellar region 3b integrations. Figure shows the sequences of the integration target sites in the *E. coli* K12 MG1655 flagellar region 3b (*fliE* (fliEi), *fliF* (fliFi), *fliG* (fliGi), *fliJ* (fliJi), *fliK* (fliKi), *fliL* (fliLi), *fliM* (fliMi), *fliP* (fliPi). The exact positions within the target genes where the integrations occurred are highlighted with stars.

**Fig 5:**
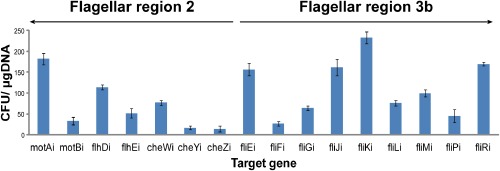
High integration efficiency into the *E. coli* flagellar region 3b. Figure showing the integration efficiency (CFU per microgram of electroporated DNA) into the investigated target loci of the *E. coli* K12 MG1655 flagellar regions 2 [*motA* (motAi), *motB* (motBi), *flhD* (flhDi), *flhE* (flhEi), *cheW* (cheWi), *cheY* (cheYi) and *cheZ* (cheZi)] and 3b [*fliE* (fliEi), *fliF* (fliFi), *fliG* (fliGi), *fliJ* (fliJi), *fliK* (fliKi), *fliL* (fliLi), *fliM* (fliMi), *fliP* (fliPi) and *fliR* (fliRi)]. The figure shows means and standard deviations from three experiments.

As *E. coli* chromosomal loci differ in their ability to support integration of genetic circuits (Juhas *et al*., 2014b), we investigated the integration efficiency for each of the 16 target loci. Genetic circuit was integrated into the investigated target sites [*motA* (motAi), *motB* (motBi), *flhD* (flhDi), *flhE* (fhlEi), *cheW* (cheWi), *cheY* (cheYi), *cheZ* (cheZi)], *fliE* (fliEi), *fliF* (fliFi), *fliG* (fliGi), *fliJ* (fliJi), *fliK* (fliKi), *fliL* (fliLi), *fliM* (fliMi), *fliP* (fliPi) and *fliR* (fliRi)] (Figs 3 and 4) and the integration efficiency was determined from the number of colony-forming units per microgram of electroporated DNA. Chromosomal integrations were confirmed with polymerase chain reaction (PCR) using flanking primers (Fig. S1) and sequencing. The primers used for the integration of the genetic circuit into the chromosome and the flanking primers are listed in Table 3. The investigated target loci differed significantly in their suitability to support integration of the genetic circuit. From the analysed genes of the *E. coli* K12 MG1655 flagellar region 2, the integration efficiency into *motA* (motAi) was highest (Fig. 5). From the *E. coli* K12 MG1655 flagellar region 3b, *fliK* (fliKi) supports the highest integration efficiency (Fig. 5). Notably, integrations into one and four loci of the flagellar regions 2 (motAi) and 3b (fliEi, fliJi, fliKi, fliRi), respectively, occurred with the higher efficiency than integrations into the previously examined flagellar regions 3a (Juhas *et al*., 2014b) and 1 (Juhas and Ajioka, 2015). Furthermore, integration efficiency into *fliK* (fliKi) was significantly higher than that of *motA* (motAi) (Fig. 5). Hence, the *E. coli* K12 MG1655 flagellar region 3b supports the highest efficiency of integration of all *E. coli* flagellar regions.

**Table 3:** Primers used in this study

Primer (Sequence 5′→ 3′)
motArepF: *motA* integration primer forward
CTCCAAATACACCAAAGCAATGTATATGGATCTGCTGGCTCTGCTTTATCGGTTGATGGCGAAATCGTATCACGAGGCAGAATTTCAGAT
motArepR: *motA* integration primer reverse
TTTCTGACGTAAAACAGTCGCTAATGGGGAAATAAATCCGTAAGCCAATAAAATGCCGAGGAAAGTCGGTTTTAAAGAAAAAGGGCAGG
motBrepF: *motB* integration primer forward
ACATGGATCGTGGAAGATTGCTTATGCCGACTTTATGACTGCGATGATGGCCTTTTTTCTGGTGATGTATCACGAGGCAGAATTTCAGAT
motBrepR: *motB* integration primer reverse
AATTTCCGCAATCGACTTTGCTCCATGCGTTTTTTCAGCTCTTCGATGTTCGGCTGCTTATTCACTCGGTTTTAAAGAAAAAGGGCAGG
flhDrepF: *flhD* integration primer forward
ATGCATACCTCCGAGTTGCTGAAACACATTTATGACATCAACTTGTCATATTTACTACTTGCACAGGTATCACGAGGCAGAATTTCAGAT
flhDrepR: *flhD* integration primer reverse
CTTCTTCAGGCTGATTAACATCATTCAGCAAGCGTGTTGAGAGCATGATGCCGGTATGAATTTGCTCGGTTTTAAAGAAAAAGGGCAGG
flhErepF: *flhE* integration primer forward
CAATTGGCGGCAAATAATGAGAACCTTATTAGCAATATTATTGTTTCCGCTGCTGGTGCAAGCCGGTATCACGAGGCAGAATTTCAGAT
flhErepR: *flhE* integration primer reverse
TTGTCCTTCAGCGATAATTCACAATCACTTCATTACGTTGTACCTTTAGCGGTGGAATTAACCGCCCGGTTTTAAAGAAAAAGGGCAGG
cheWrepF: *cheW* integration primer forward
TACCCTTGGTGATGAAGAGTACGGTATTGATATCCTGAAAGTGCAGGAGATCCGTGGCTACGATCAGTATCACGAGGCAGAATTTCAGAT
cheWrepR: *cheW* integration primer reverse
TATCTAACAGCGCCATCTCTTCGCTGTTCAGCAGTTTTTCGATGTTCACCAGAATCAACATCCGGTCGGTTTTAAAGAAAAAGGGCAGG
cheYrepF: *cheY* integration primer forward
AAAGAGCTGGGATTCAATAATGTTGAGGAAGCGGAAGATGGCGTCGACGCTCTCAATAAGTTGCAGGTATCACGAGGCAGAATTTCAGAT
cheYrepR: *cheY* integration primer reverse
AGTTTCTCAAAGATTTTGTTGAGTTTTTCCTCCAGCGTCGCGGCGGTAAATGGCTTCACCACATAGCGGTTTTAAAGAAAAAGGGCAGG
cheZrepF: *cheZ* integration primer forward
CAGGATTTTCAGGATCTCACCGGGCAGGTCATTAAGCGGATGATGGATGTCATTCAGGAGATCGAAGTATCACGAGGCAGAATTTCAGAT
cheZrepR: *cheZ* integration primer reverse
TCAAAATCCAAGACTATCCAACAAATCGTCCACCTGATCCTGACTGGCTACCACACCGGCTTTGCTCGGTTTTAAAGAAAAAGGGCAGG
motArepTF: *motA* integration test primer forward
GCGCTGCCGTTGCTGTTTCG
motArepTR: *motA* integration test primer reverse
GCAGAGTGACTTTGACGCACTGCA
motBrepTF: *motB* integration test primer forward
CGTCAAACGACGCAAAGCCAAAAGC
motBrepTR: *motB* integration test primer reverse
GACGTAACGCCCGCAGTTTCG
flhDrepTF: *flhD* integration test primer forward
GCTTCCCGGCGACATCACG
flhDrepTR: *flhD* integration test primer reverse
AGGCCCTTTTCTTGCGCAGC
flhErepTF: *flhE* integration test primer forward
CTGTCTGATAACCGACATATCCGCATGACG
flhErepTR: *flhE* integration test primer reverse
CACTGAGTTATTAAACATACTCGCGAGCGC
cheWrepTF: *cheW* integration test primer forward
ACCGCCGCCTGAATGAGTAAAAAGG
cheWrepTR: *cheW* integration test primer reverse
CGGGAGAATTACGCCACTTCTGACG
cheYrepTF: *cheY* integration test primer forward
CCACCATGCGACGCATAGTGC
cheYrepTR: *cheY* integration test primer reverse
GCATCATAGTCGCATCCTCACATGCC
cheZrepTF: *cheZ* integration test primer forward
CACGACAATTTCTGGCAGATGTACCCG
cheZrepTR: *cheZ* integration test primer reverse
TCAGACCGCCTGATATGACGTGGT
fliEreprF: *fliE* integration primer forward
GTTAAACGATGTGATGACCGATATGCAAAAAGCCTCAGTTTCTATGCAAATGGGGATTCAGGTGCGGTATCACGAGGCAGAATTTCAGAT
fliEreprR: *fliE* integration primer reverse
GGATCGTGGATGGCAGAAAACGTTCAGGATCAGGTATCCATTTTAAACCAGAAATTGAGTGAGTTTCGGTTTTAAAGAAAAAGGGCAGG
fliFreprF: *fliF* integration primer forward
ATTATTCAGCAATCTTTCCGATCAGGATGGTGGCGCAATTGTCAGCCAACTGACGCAAATGAATATGTATCACGAGGCAGAATTTCAGAT
fliFreprR: *fliF* integration primer reverse
CTTCAATACGCCGCTGAATACGGCCTTCGACATCGCTGGCATATTTCAACTGAGCGTCATTAAGATCGGTTTTAAAGAAAAAGGGCAGG
fliGreprF: *fliG* integration primer forward
GCCGCACTGAATATCAACGCCAACGATTATCTGCGCTCGGTATTGGTCAAAGCTCTGGGTGAAGAAGTATCACGAGGCAGAATTTCAGAT
fliGreprR: *fliG* integration primer reverse
CGCTGAATGCTGCGATCGTCGACATCCACCAGATTCTCGAACAGGAACATCTCGTCGATGATTTTCCGGTTTTAAAGAAAAAGGGCAGG
fliJreprF: *fliJ* integration primer forward
GAAATGCGTCGCGGATGTCAGCAGGCGGAAGAACAGCTCAAAATGCTGATTGATTATCAGAATGAAGTATCACGAGGCAGAATTTCAGAT
fliJreprR: *fliJ* integration primer reverse
TGCAAACGTTGTTTTTTTTCTCGCCAACTGTTCAGGGCAATGTCAACTTTCTGCGTCCACTGATTACGGTTTTAAAGAAAAAGGGCAGG
fliKreprF: *fliK* integration primer forward
TATTGTTTCCGACGCGCAACAAGCTAATTTACTGATCCCTGTGGATGAAACACCGCCTGTCATCAAGTATCACGAGGCAGAATTTCAGAT
fliKreprR: *fliK* integration primer reverse
GTGAAACCATCTGGATTTGCGCCTGGTTATCATCCACTTTGAGGGAGATTTGCACTTCACCTAAATCGGTTTTAAAGAAAAAGGGCAGG
fliLreprF: *fliL* integration primer forward
ATGACTGATTACGCGATAAGCAAGAAAAGCAAGCGATCGCTTTGGATCCCGATTCTGGTATTCATTGTATCACGAGGCAGAATTTCAGAT
fliLreprF: *fliL* integration primer reverse
GTGGTTTTAATCTCGGCAATCAGGTTTTTCTTGCCTTCTTCTGTCGCCAGTACGGCAGCATCCTGACGGTTTTAAAGAAAAAGGGCAGG
fliMreprF: *fliM* integration primer forward
AAATTTACCAATATCACCACCTCGCCGAACGACATTGTGGTTAACACGCCGTTCCATGTGGAGATTGTATCACGAGGCAGAATTTCAGAT
fliMreprF: *fliM* integration primer reverse
AAATCGGGTTAATCAAATGTTCTATCCGTAACGCATACTGACCGTTGAGGGTGCCATACTGACTGGCGGTTTTAAAGAAAAAGGGCAGG
fliPreprF: *fliP* integration primer forward
GGTATTGCTGGGGCTGGCACTGTTTTTGACCTTTTTTATTATGTCACCGGTGATCGACAAAATTTAGTATCACGAGGCAGAATTTCAGAT
fliPreprF: *fliP* integration primer reverse
AACACGCTGGCTATCACCAGGTCGATAATCAAAAAAGGGATGAAAATCGTGAAGCCTATCTGAAATCGGTTTTAAAGAAAAAGGGCAGG
fliRreprF: *fliP* integration primer forward
TATCATGGATATGCTGGCGTTACTGCTGTTCCTGACATTTAACGGTCATTTATGGTTGATTTCACTGTATCACGAGGCAGAATTTCAGAT
fliRreprF: *fliP* integration primer reverse
ATAATATCAGCCAGCAAATTAAAAATTTCACTGAATAAATGTTCGCAAAAAGGTGCAATTAACGGCCGGTTTTAAAGAAAAAGGGCAGG
fliErepTF: *fliE* integration test primer forward
GCCGCAACCGACCATTAGTTTTGC
fliErepTR: *fliE* integration test primer reverse
GCTCTGATGTTCAGGGGGGTTATCG
fliFrepTF: *fliF* integration test primer forward
GTCATGGTCGCACTGATCCTGTGG
fliFrepTR: *fliF* integration test primer reverse
CAGCTGCGCCGTAACCTGG
fliGrepTF: *fliG* integration test primer forward
CTGGCGGAGTTTGAGCAAGAAGC
fliGrepTR: *fliG* integration test primer reverse
CGGCGTCCAGGTTTTCCACG
fliJrepTF: *fliJ* integration test primer forward
GCTGGCGACCCTGAAAGATCTGG
fliJrepTR: *fliJ* integration test primer reverse
GCCGTTCCTGCAGTGTCTGC
fliKrepTF: *fliK* integration test primer forward
GCCCACGACAAAAGGCGAGC
fliKrepTR: *fliK* integration test primer reverse
GGTTCATGGTTTGCTGTGCGTTGG
fliLrepTF: *fliL* integration test primer forward
AGCAGTAGCGACACAGGAAGACC
fliLrepTR: *fliL* integration test primer reverse
CGGTGACATCCTGTTTCGGTTGC
fliMrepTF: *fliM* integration test primer forward
GTTGAGTACGTGCGTTCGGAAATGC
fliMrepTR: *fliM* integration test primer reverse
CACTCATTTGGGCTGTTCCTCGTTCAG
fliPrepTF: *fliP* integration test primer forward
GTGGACAAAGCTGGTCGCTCC
fliPrepTR: *fliP* integration test primer reverse
GGCAGAGCAATGGTGGCTGG
fliRrepTF: *fliR* integration test primer forward
ATGGGGCTGTCATTTGCGACG
fliRrepTR: *fliR* integration test primer reverse
CAGGCGGACTTACTATCCCGTAAAGTG

### Integrations into flagellar regions 2 and 3b abolish motility

**Fig 6:**
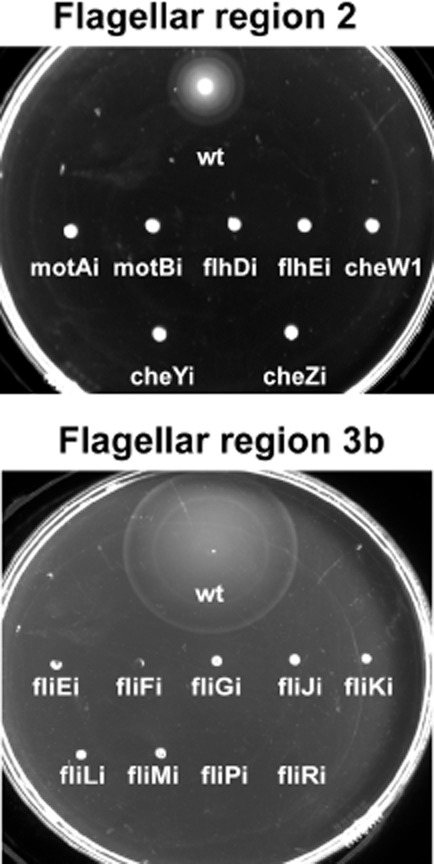
Integrations into the *E. coli* flagellar regions 2 and 3b abolish motility. The integrations into the investigated target sites of the flagellar region 2 [*motA* (motAi), *motB* (motBi), *flhD* (flhDi), *flhE* (flhEi), *cheW* (cheWi), *cheY* (cheYi) and *cheZ* (cheZi)] and 3b [*fliE* (fliEi), *fliF* (fliFi), *fliG* (fliGi), *fliJ* (fliJi), *fliK* (fliKi), *fliL* (fliLi), *fliM* (fliMi), *fliP* (fliPi)] completely abolished the motility of the engineered strains. Wt (*E. coli* K12 MG1655 wild type). *E. coli* cultures normalized to OD_600_ of 1 were inoculated in the motility agar plates and the picture was taken after 5 h of incubation at 37°C.

Flagellum is crucial for the motility of *E. coli* cells. Therefore, the disruptions of the flagellar functions-encoding genes usually have a negatively impact on motility (Juhas *et al*., 2014b). Integrations into two genes of the previously analysed flagellar region 3a only reduced motility of the engineered strains when compared with the wild type (Juhas *et al*., 2014b). We investigated the effect of the chromosomal integrations into the flagellar regions 2 and 3b by spotting 2 μl of the normalized overnight cultures of the engineered *E. coli* strains and *E. coli* K12 MG1655 wild type in the middle of the motility agar plates (Fig. 6). The motility of all strains harbouring integrations in the investigated genes of the flagellar regions 2 (Fig. 6A) and 3b (Fig. 6B) was completely abolished.

### Integrations into flagellar regions do not have negative impact on the growth

As integrations of the synthetic genetic circuits into the *E. coli* chromosome should not negatively impact cell growth, target loci cannot be located within essential genes (Juhas *et al*., 2011; 2012a,b; 2014a). To assess the effect of chromosomal integrations into the seven investigated genes of the flagellar regions 2 (*motA*, *motB*, *flhD*, *flhE*, *cheW*, *cheY* and *cheZ*) and 3b (*fliE*, *fliF*, *fliG*, *fliJ*, *fliK*, *fliL*, *fliM*, *fliP* and *fliR*) on the growth rate, the absorbance of the engineered strains and K12 MG1655 wild type was measured with the microplate reader (Fluostar Omega). Integrations into all investigated genes of the flagellar regions 2 (Fig. S2) and 3b (Fig. S3) did not diminish growth rate when compared with the wild type at both 30°C and 37°C. This is consistent with previous results from flagellar regions 3a (Juhas *et al*., 2014b) and 1 (Juhas and Ajioka, 2015).

### Transcription of the flagellar regions 2 and 3b

**Fig 7:**
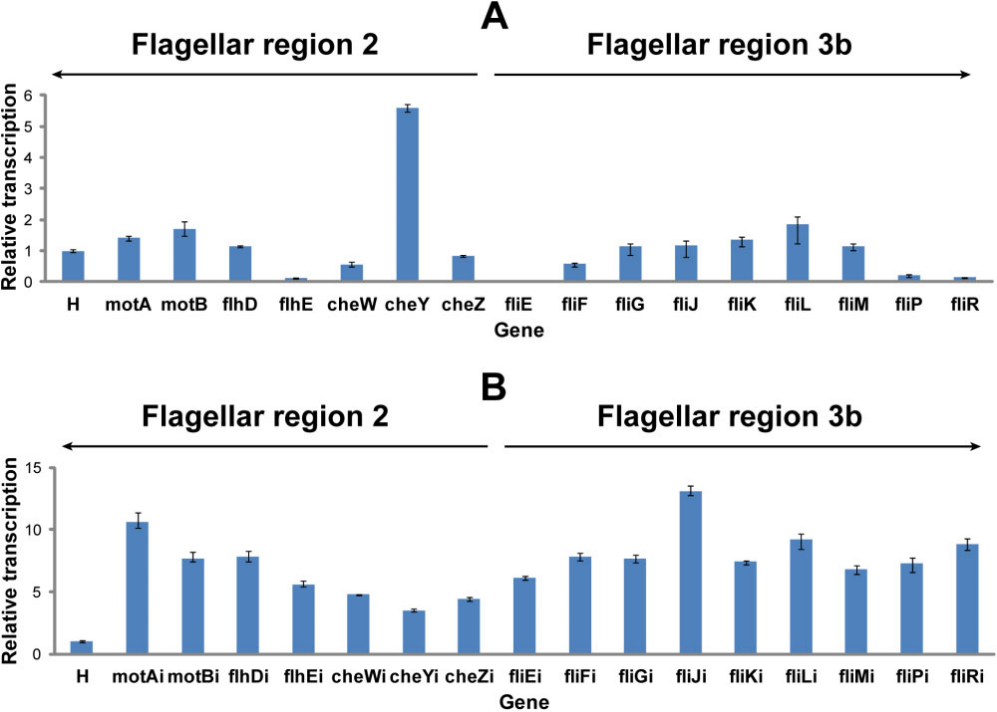
RT-PCR analysis.A. Relative transcription of the analysed target genes of the *E. coli* K12 MG1655 flagellar regions 2 (*motA*, *motB*, *flhD*, *flhE*, *cheW*, *cheY* and *cheZ*) and 3b (*fliE*, *fliF*, *fliG*, *fliJ*, *fliK*, *fliL*, *fliM*, *fliP*, *fliR*) compared with the housekeeping genes (H).B. Transcription of the genetic circuit integrated in the investigated integration target loci of the *E. coli* flagellar region 2 [*motA* (motAi), *motB* (motBi), *flhD* (flhDi), *flhE* (flhEi), *cheW* (cheWi), *cheY* (cheYi) and *cheZ* (cheZi)] and flagellar region 3b [(*fliE* (fliEi), *fliF* (fliFi), *fliG* (fliGi), *fliJ* (fliJi), *fliK* (fliKi), *fliL* (fliLi), *fliM* (fliMi), *fliP* (fliPi) and *fliR* (fliRi)] relative to the transcription of the housekeeping genes (H). Bars and errors represent averages and standard errors from three experiments. H (mean transcription of the reference housekeeping genes *arcA* and *rpoD*). Relative transcription was quantified with rest9 Software (Qiagen) employing Pfaffl method (Pfaffl *et al*., 2002).

The relative transcription of the investigated genes of the flagellar regions 2 and 3b was measured by real-time polymerase chain reaction (RT-PCR) using *arcA* and *rpoD* as the reference housekeeping genes (Jandu *et al*., 2009; Minty *et al*., 2011). Real-time polymerase chain reaction (RT-PCR) showed that the relative expression of four genes from both analysed flagellar regions 2 (*motA*, *motB*, *flhD*, *cheY*) and 3b (*fliJ*, *fliK*, *fliL*, *fliM*) was higher (twofold to fivefold) than the average expression of the housekeeping genes (Fig. 7A). The relative transcription of *fliG* was not significantly different, whereas the transcription of the remaining genes was lower than the mean expression of the housekeeping genes (Fig. 7A). The transcription of the genetic circuit integrated into *motA* (motAi), *motB* (motBi), *flhD* (flhDi), *flhE* (fhlEi), *cheW* (cheWi), *cheY* (cheYi) and *cheZ* (cheZi) of the flagellar region 2 and *fliE* (fliEi), *fliF* (fliFi), *fliG* (fliGi), *fliJ* (fliJi), *fliK* (fliKi), *fliL* (fliLi), *fliM* (fliMi), *fliP* (fliPi) and *fliR* (fliRi) of the flagellar region 3b measured by RT-PCR was high at all analysed loci (Fig. 7B). From the flagellar region 2, highest expressed (8- to 11-fold higher than the housekeeping genes) was the genetic circuit integrated into *motA* (motAi), *motB* (motBi) and *flhD* (flhDi) (Fig. 7B). The expression at *flhE* (fhlEi), *cheW* (cheWi) and *cheY* (cheYi) was fourfold to sixfold higher than the mean expression of the housekeeping genes (Fig. 7B). From the flagellar region 3b, highest expressed (8- to 13-fold higher than the housekeeping genes) was the genetic circuit integrated into *fliJ* (fliJAi), *fliL* (fliLi) and *fliR* (fliRi) (Fig. 7B). The expression at the remaining loci of the flagellar region 3b was sixfold to eightfold higher than the mean expression of the housekeeping genes (Fig. 7B). Such strong expression of the genetic circuit integrated into this flagellar region is interesting, particularly when considering that the flagellar region 3b shows lowest probability of being occupied by RNA polymerase (Fig. 1B). This suggests that other factors might be also important for the expression of the integrated synthetic DNA and shows that empirical characterization is necessary for engineering into integration sites. Expression of the integrated genetic circuit was determined by the quantitative measurement of the green fluorescent protein (GFP) and the red fluorescent protein (mCherry) fluorescence over time with the microplate reader (FLUOstar Omega). For this, we have used plasmids pSB1A1(GFP) and pSB1A1(mCh) harbouring GFP and mCherry, respectively, regulated by the pR promoter. Both GFP and mCherry were not expressed at permissive conditions for the repressor (30°C), while the temperature shift to 42°C set off GFP and mCherry expression (Figs S4–S7).

### Conclusions

Flagellar regions are good targets for integration of genetic circuits into the *E. coli* chromosome. The identification of reliable target loci is crucial for building a robust synthetic biology toolkit and for *E. coli* bioengineering. Furthermore, it can be exploited for tighter regulation of expression of toxic proteins in *E. coli*. In this study, we have integrated genetic circuit into 16 well-conserved open reading frames of the *E. coli* K12 MG1655 flagellar regions 2 (*motA*, *motB*, *flhD*, *flhE*, *cheW*, *cheY* and *cheZ*) and 3b (*fliE*, *fliF*, *fliG*, *fliJ*, *fliK*, *fliL*, *fliM*, *fliP* and *fliR*). The integrations into all target loci of these flagellar regions led to the loss of motility, but did not reduce the growth rate of the engineered strains. *Escherichia coli* K12 MG1655 flagellar region 3b supports highest efficiency of integration of all *E. coli* flagellar regions. Notably, the genetic circuit integrated into flagellar region 3b was also highly expressed although the probability of the RNA polymerase binding into this region is significantly lower than into other flagellar regions. This suggests that other factors might also play a role in the expression of the integrated synthetic DNA. There appears to be a weak inverse correlation between the probability of RNA polymerase binding to the target loci and their ability to support integration of the genetic circuit; however, this will require further investigation. Furthermore, as flagellar genes are closer to the terminal (TER) region of the *E. coli* chromosome than oriC, their copy number is approximately sixfold lower than those genes close to oriC during exponential growth. Therefore, genes nearer to oriC are also potentially interesting target loci for integration and expression of genetic circuits. Besides the modified lambda Red recombinase method used in our analysis, clustered regularly interspaced short palindromic repeats (CRISPR) and integrases could be exploited for *E. coli* engineering. A variety of high complexity integrase sites, such as phiC31, R4 and Bxb1, could be moved to the hotspot integration regions in the *E. coli* chromosome employing CRISPR for appending new functionalities. Overall, the *E. coli* K12 MG1655 flagellar region 3b is the most suitable of all *E. coli* flagellar regions for integration and expression of genetic circuits. However, there is a significant variation between individual target loci. For instance, *motA* of the *E. coli* K12 MG1655 flagellar region 2 supports the second highest integration and expression efficiency of all investigated target sites in this study (Figs 5 and 7). Therefore, when considered individually, *fliJ* and *motA* appear to be the most suitable integration target loci of the analysed flagellar regions 2 and 3b.

## Experimental procedures

### Bacterial strains, plasmids and growth conditions

All strains and plasmids used in this study are recorded in Table 4. *Escherichia coli* was routinely grown in Luria–Bertani (LB) medium supplemented with ampicillin (100 μg ml^−1^) or kanamycin (50 μg ml^−1^) when required. Liquid *E. coli* cultures were cultivated on a rotatory shaker at 200 r.p.m. at 30°C, 37°C or 42°C. Plate cultures were supplemented with 1% agar (w/v) and grown for about 24 h at 30°C, 37°C or 42°C.

**Table 4:** Bacterial strains and plasmids

	Characteristics	Reference
Strains		
K12 MG1655	*E. coli* wild type	Hayashi *et al*., 2006
Ec:motAi	*E. coli* K12 MG1655, *motA* integration	This study
Ec:motBi	*E. coli* K12 MG1655, *motB* integration	This study
Ec:flhDi	*E. coli* K12 MG1655, *flhD* integration	This study
Ec:flhEi	*E. coli* K12 MG1655, *flhE* integration	This study
Ec:cheWi	*E. coli* K12 MG1655, *cheW* integration	This study
Ec:cheYi	*E. coli* K12 MG1655, *cheY* integration	This study
Ec:cheZi	*E. coli* K12 MG1655, *cheZ* integration	This study
Ec:fliEi	*E. coli* K12 MG1655 integration into *fliE*	This study
Ec:fliFi	*E. coli* K12 MG1655 integration into *fliF*	This study
Ec:fliGi	*E. coli* K12 MG1655 integration into *fliG*	This study
Ec:fliJi	*E. coli* K12 MG1655 integration into *fliJ*	This study
Ec:fliK	*E. coli* K12 MG1655 integration into *fliK*	This study
Ec:fliLi	*E. coli* K12 MG1655 integration into *fliL*	This study
Ec:fliMi	*E. coli* K12 MG1655 integration into *fliM*	This study
Ec:fliPi	*E. coli* K12 MG1655 integration into *fliP*	This study
Ec:fliRi	*E. coli* K12 MG1655 integration into *fliR*	This study
Plasmids		
pCP20	Plasmid encoding FLP recombinase	Datsenko and Wanner, 2000
pKM208	IPTG-induced Red recombinase system	Murphy and Campellone, 2003
pSB1A1(GFP)	λ promoter-controlled GFP, Amp^R^	Juhas *et al*., 2014b
pSB1A1(mCh)	λ promoter-controlled mCherry, Amp^R^	This study
pSB1K3(FRTKr)	λ repressor, Kan^R^	Juhas *et al*., 2014b

### DNA amplification and modification

DNA was amplified by PCR in 50 μl of reaction volumes employing Phusion DNA polymerase (Thermo Scientific) or Dream Taq master mix kit (Thermo Scientific) according to the supplier's instructions. Oligonucleotide primers for PCR amplifications were synthesized by Integrated DNA Technologies (IDT) and Sigma-Aldrich. DNA fragments were purified by gel electrophoresis, followed by gel extraction employing Qiaquick Gel Extraction kit (Qiagen), according to the manufacturer's instructions. Plasmid DNA was performed with the Qiaprep Spin Miniprep kit (Qiagen), according to the supplier's recommendations. Sequencing was performed by Source Bioscience (Cambridge, UK). A Gibson Isothermal Assembly method (Gibson *et al*., 2009; Merryman and Gibson, 2012) was employed to assemble DNA fragments. The original Gibson Isothermal Assembly method protocol was modified as described previously (Juhas *et al*., 2014b).

### Integration of the genetic circuit into the chromosome

Altered Hannah (Hanahan *et al*., 1991) and Miller and Nickoloff (1995) protocols were used to prepare the chemically competent and electro-competent *E. coli* cells respectively. Integrations of the genetic circuit into target open reading frames of the analysed *E. coli* flagellar region were carried out using method described previously (Juhas *et al*., 2014b). Briefly, plasmid pKM208 was transformed into the wild-type *E. coli* K12 MG1655 and selected on plates with ampicillin at 30°C. *Escherichia coli* K12 MG1655 harbouring pKM208 was inoculated into LB with ampicillin and grown at 30°C. After reaching OD_600_ of 0.2, 1 mM IPTG was added and the bacterial culture was cultivated to the final OD_600_ of 0.4–0.6. Bacteria were subsequently washed and resuspended in 10% glycerol and transformed with the genetic circuit harbouring the flanking sequences of the target genes. Bacteria with chromosomal integrations were selected on plates with kanamycin at 37°C and subsequently grown at 42°C to cure out the temperature-sensitive plasmid pKM208. Chromosomal integrations were proved by PCR with flanking primers and sequencing.

### GFP and mCherry fluorescence quantitation with the microplate reader

200 μl of the *E. coli* cultures (grown overnight and diluted to OD_600_ of 0.05) were transferred into flat-bottomed black 96 well plates (Greiner BioOne, UK). The plates with the *E. coli* cultures were placed into Fluostar Omega fluorimeter (BMG Labtech, UK) and incubated first at 30°C for 3 h and then at 42°C for 17 h or 7 h for GFP and mCherry fluorescence measurement respectively. GFP fluorescence was quantified with an automatically repeated protocol each 30 min using emission filter EM520, excitation filter 485-12, double orbital shaking at 200 r.p.m. and gain 1400. mCherry fluorescence was measured with an automatically repeated protocol each 30 min using emission filter EM620, excitation filter 584, double orbital shaking at 200 r.p.m. and gain 2800.

### Absorbance measurement with the microplate reader

The diluted overnight *E. coli* cultures (OD_600_ of 0.05) were transferred into flat-bottomed clear 96 well plates (Sterilin Sero-Well, UK). The plates were then incubated in the microplate reader (Fluostar Omega, BMG Labtech, UK) at 37°C and 30°C for 24 h. Absorbance was measured each 30 min using 600 nm absorbance filter and double orbital shaking at 500 r.p.m.

### RNA isolation and purification

Total RNA was isolated from 10^9^ *E. coli* cells at mid-exponential phase with Isolate II RNA Mini Kit (Bioline) according to manufacturer's instructions. To elute RNA from the Isolate II RNA columns, 60 μl of RNAse-free H_2_O was used. To avoid contamination with genomic DNA, the isolated RNA was purified with TURBO DNA-free Kit (Applied Biosystems) according to supplier's instructions.

### RT-PCR

Isolated and purified RNA (1 μg) was used to synthesize cDNA using SuperScript III Reverse Transcriptase (Invitrogen) according to supplier's instructions. Primers for RT-PCR designed with primer3 Software were prepared to generate 100–150 bp long DNA sequences. Expression levels were measured using QuantiTect SYBR Green PCR Kit (Qiagen). MicroAmp Fast Optical 96-Well Reaction Plates (Applied Biosystems) with RT-PCR reactions were incubated in the 7500 Fast Real-Time PCR System (Applied Biosystems) according to manufacturer's instructions. The relative expression was computed employing rest9 Software (Qiagen) with Pfaffl method (Pfaffl *et al*., 2002). The RT-PCR was performed in triplicate, and the means and standard errors were calculated.

### Evaluation of motility

Motility agar plates for motility assay were made by transferring 100 ml of motility agar [composed of 0.25% Bacto-Agar (Difco), 5 g NaCl and 10 g tryptone] in the 13 cm plates and let to set overnight. Plates were then pre-warmed to 37°C and inoculated with the 2 μl of the overnight bacterial cultures normalized to OD_600_ of 1.0. Pictures were taken after incubation for 4–6 h at 37°C.

### Sequence analyses

The annotated *E. coli* K-12 MG1655 genome from the *E. coli* K-12 project website (http://www.xbase.ac.uk/genome/escherichia-coli-str-k-12-substr-mg1655) was used to retrieve DNA sequences of the target loci. DNA sequencing was carried out by Source Bioscience (Cambridge, UK). The blastn (Altschul *et al*., 1990) and tblastx algorithms from the National Center for Biotechnology Information (NCBI) website (http://ncbi.nlm.nih.gov) and the position-specific iterated blast (PSI-BLAST) (Altschul *et al*., 1997) were used to compare DNA sequences.

## Supporting Information

**Fig. S1.** Verification of chromosomal integrations into the *E. coli* flagellar region 2. **Fig. S2.** Growth rates of the engineered *E. coli* strains with integrations in the flagellar region 2. **Fig. S3.** Integrations into the *E. coli* flagellar region 3b do not negatively impact growth. **Fig. S4.** Fluorescence measurement of the *E. coli* flagellar region 2 integrations. **Fig. S5.** Fluorescence measurement of the *E. coli* flagellar region 3b integrations. **Fig. S6.** mCherry fluorescence quantitation of the *E. coli* flagellar region 2 integrations. **Fig. S7.** mCherry fluorescence quantitation of the *E. coli* flagellar region 3b integrations.
