# Supplementary material for: Flagellar region 3b supports strong expression of integrated DNA and the highest chromosomal integration efficiency of the Escherichia coli flagellar regions
Source: Microb Biotechnol. 2015 Jun 15;8(4):726–38. doi: 10.1111/1751-7915.12296 (PMC4476827; doi:10.1111/1751-7915.12296)
Supplement: Supplementary file 1 — Fig. S1. Verification of chromosomal integrations into the E. coli flagellar region 2. Fig. S2. Growth rates of the engineered E. coli strains with integrations in the flagellar region 2. Fig. S3. Integrations into the E. coli flagellar region 3b do not negatively impact growth. Fig. S4. Fluorescence measurement of the E. coli flagellar region 2 integrations. Fig. S5. Fluorescence measurement of the E. coli flagellar region 3b integrations. Fig. S6. mCherry fluorescence quantitation of the E. coli flagellar region 2 integrations. Fig. S7. mCherry fluorescence quantitation of the E. coli flagellar region 3b integrations. [file mbt20008-0726-sd1.pdf]

Supporting Information

S1 Fig. Verification of chromosomal integrations into the *E. coli* flagellar region 2.

Chromosomal integrations into the target genes of the *E. coli* K12 MG1655 flagellar region 2 (*motA* (motAi), *motB* (motBi), *flhD* (flhDi), *flhE* (flhEi), *cheW* (cheWi), *cheY* (cheYi), and *cheZ* (cheZi)) verified by PCR with flanking primers. Wt (wild type), +i (integrated DNA fragment). HyperLadder 1kb (Bioline) has been used as the molecular weight marker.

(TIFF)

S2 Fig. Growth rates of the engineered *E. coli* strains with integrations in the flagellar region 2.

Growth rates (measured as absorbance over time with the microplate reader) of the engineered strains with integrations in the investigated target loci of the flagellar region 2 (*motA* (motAi), *motB* (motBi), *flhD* (flhDi), *flhE* (flhEi), *cheW* (cheWi), *cheY* (cheYi), and *cheZ* (cheZi)) compared to the *E. coli* K12 MG1655 wild type (wt). Values represent averages and standard errors from three independent replicates. Raw absorbance plate reader data are shown in the S2 Table.

S3 Fig. Integrations into the *E. coli* flagellar region 3b do not negatively impact growth.

Microplate reader ((Fluostar Omega) absorbance measurement of the growth of the engineered strains with integrations into the target loci of the *E. coli* K12 MG1655 flagellar region 3b (*fliE* (fliEi), *fliF* (fliFi), *fliG* (fliGi), *fliJ* (fliJi), *fliK* (fliKi), *fliL* (fliLi), *fliM* (fliMi), *fliP* (fliPi), and *fliR* (fliRi)). The figure shows means and standard errors from three experiments.

S4 Fig. GFP fluorescence measurement of the *E. coli* flagellar region 2 integrations. Figure shows verification of the integration of the genetic circuit Repr-ts-1 into the target loci of the flagellar region 2 (*motA* (motAi), *motB* (motBi), *flhD* (flhDi), *flhE* (flhEi), *cheW* (cheWi), *cheY* (cheYi), and *cheZ* (cheZi)) by quantification of the GFP fluorescence over time with the Fluostar Omega fluorimeter. Temperature shift to 42 °C (grey dashed line) after 3 hours of growth at 30 °C led to the expression of GFP in the *E. coli* strains harbouring integrations in the target loci. Fluorescence signal saturated the fluorimeter detector (260000) after 5 hours of growth in the control strain without the repressor (wt + GFP). Wt (*E. coli* K12 MG1655 wild type). Values represent averages and standard errors from three independent experiments.

S5 Fig. GFP fluorescence measurement of the *E. coli* flagellar region 3b integrations. Chromosomal integrations of the genetic circuit Repr-ts-1 into the target loci of the *E. coli* K12 MG1655 flagellar region 3b (*fliE* (fliEi), *fliF* (fliFi), *fliG* (fliGi), *fliJ* (fliJi), *fliK* (fliKi), *fliL* (fliLi), *fliM* (fliMi), *fliP* (fliPi), and *fliR* (fliRi)) were confirmed by measuring GFP fluorescence over time employing microplate reader (Fluostar Omega). The temperature shift from 30 °C to 42 °C (grey dashed line) after 3 hours of growth triggered Repr-ts-1 controlled GFP expression in the engineered strains with the chromosomal integrations in the target loci of the flagellar region 3b. GFP fluorescence was off the chart (260000) after 5 hours of growth in the positive control strain (wt + GFP). Wt (*E. coli* K12 MG1655 wild type). fliEi, Fi, Gi, Ji, Ki, Li, Mi, Pi, Ri (engineered *E. coli* K12 MG1655 strains with chromosomal integrations in the analysed genes of the flagellar region 3b harbouring pR promoter controlled GFP-expressing plasmid). wt + GFP (positive control strain harbouring pR promoter controlled GFP-expressing plasmid without the integrated the Repr-ts-1-bourne repressor).

S6 Fig. mCherry fluorescence quantitation of the *E. coli* flagellar region 2 integrations. Figure depicts confirmation of the integration of the genetic circuit into the target sites of the flagellar region 2 (*motA* (motAi), *motB* (motBi), *flhD* (flhDi), *flhE* (flhEi), *cheW* (cheWi), *cheY* (cheYi), and *cheZ* (cheZi)) by measuring the mCherry fluorescence with the Fluostar Omega fluorimeter. Temperature shift to 42 °C (grey dashed line) after 3 hours of growth at 30 °C led to the expression of mCherry in the *E. coli* with integrations in the target sites. Fluorescence signal saturated the fluorimeter detector after 5 hours of growth in the control strain without the repressor (wt + mCherry). Wt (*E. coli* K12 MG1655 wild type). Values represent averages and standard errors from three replicates.

S7 Fig. mCherry fluorescence quantitation of the *E. coli* flagellar region 3b integrations. Integrations into the target sites of the *E. coli* K12 MG1655 flagellar region 3b (*fliE* (fliEi), *fliF* (fliFi), *fliG* (fliGi), *fliJ* (fliJi), *fliK* (fliKi), *fliL* (fliLi), *fliM* (fliMi), *fliP* (fliPi), and *fliR* (fliRi)) were confirmed by measuring mCherry fluorescence over time with the microplate reader (Fluostar Omega). The temperature shift from 30 °C to 42 °C (grey dashed line) after 3 hours of growth triggered expression of the red fluorescent protein mCherry in the strains with integrations in the target sites of the flagellar region 3b. mCherry fluorescence was off the chart after 5 hours of growth in the positive control strain (wt + mCherry). Wt (*E. coli* K12 MG1655 wild type). fliEi, Fi, Gi, Ji, Ki, Li, Mi, Pi, Ri (*E. coli* K12 MG1655 with integrations in the analysed loci of the flagellar region 3b harbouring plasmid pSB1A1(mCh). wt + mCherry (positive control strain harbouring pSB1A1(mCh) without the integrated Repr-ts-1 repressor).

S1 Fig.

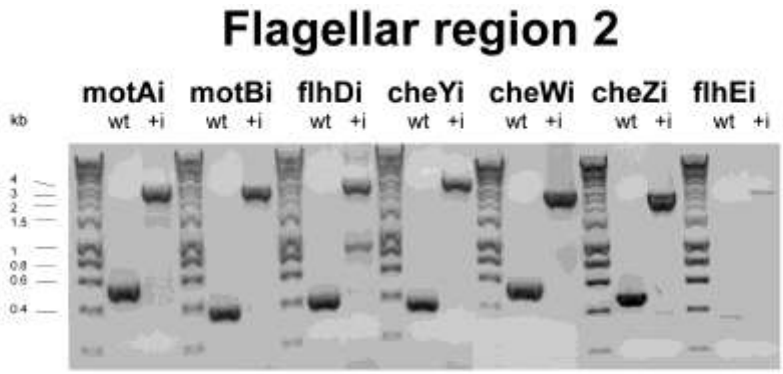
Flagellar region 2
motAi
motBi
flhDi
cheYi
cheWi
cheZi
flhEi
kb
wt +i
4
3
2
1.5
1
0.8
0.6
0.4

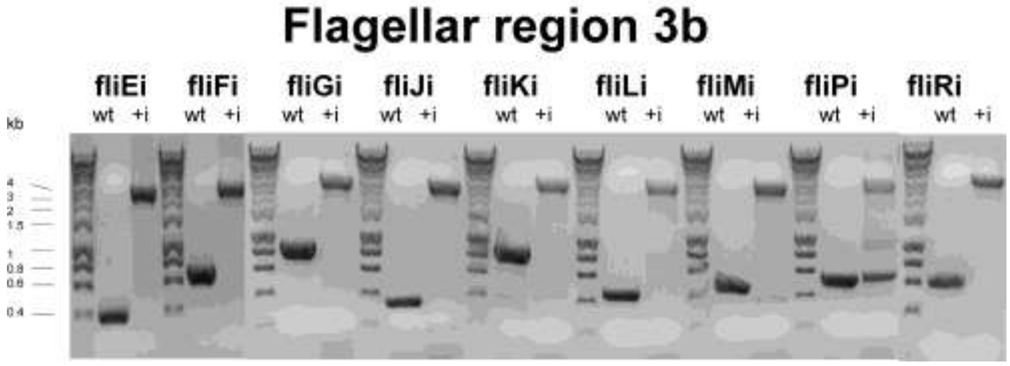
Flagellar region 3b
fliEi
fliFi
fliGi
fliJi
fliKi
fliLi
fliMi
fliPi
fliRi
kb
wt +i
4
3
2
1.5
1
0.8
0.6
0.4

S2 Fig.

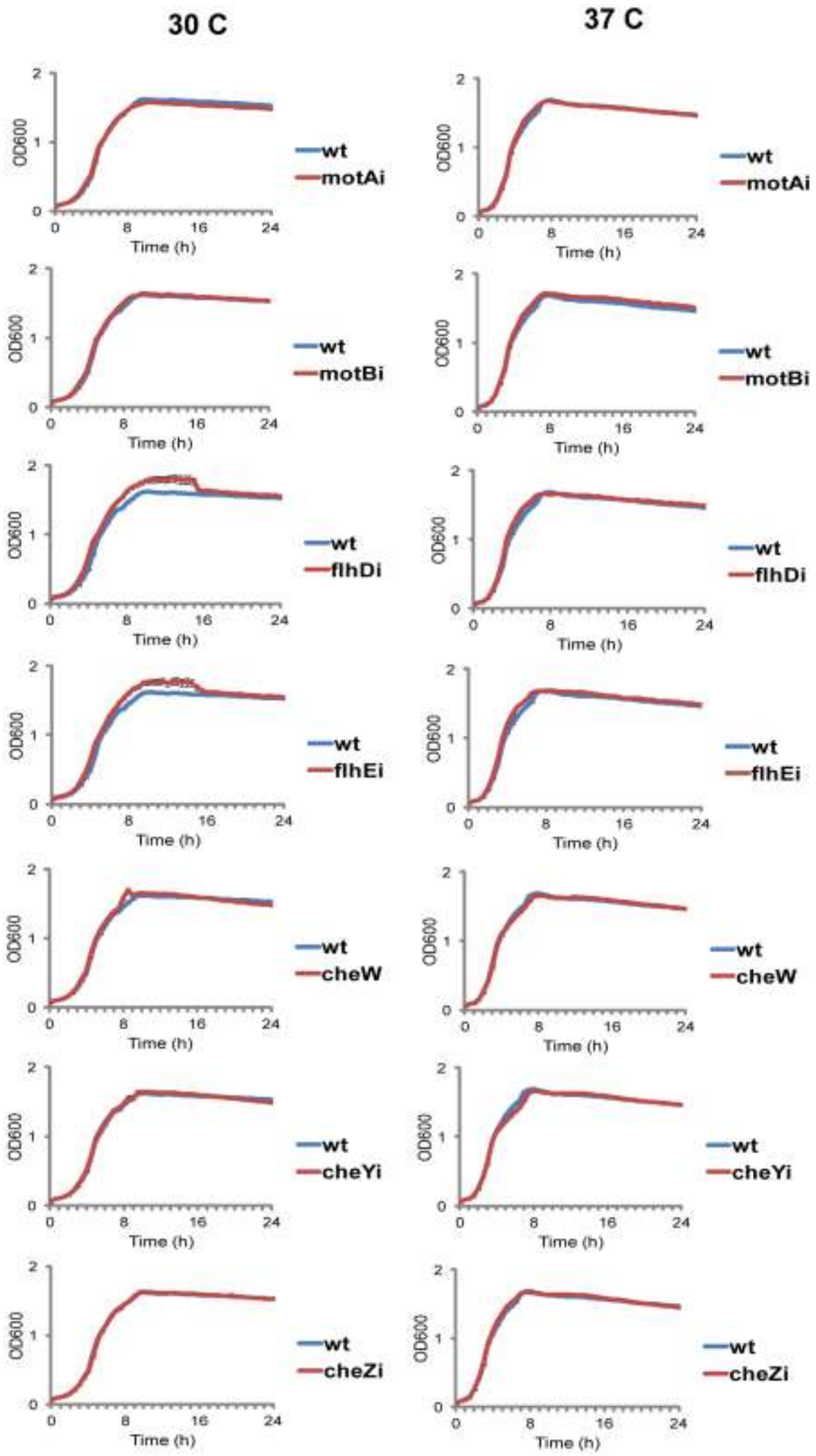
30 C
37 C
OD600
Time (h)
wt
motAi
motBi
flhDi
flhEi
cheW
cheYi
cheZi

S3 Fig.

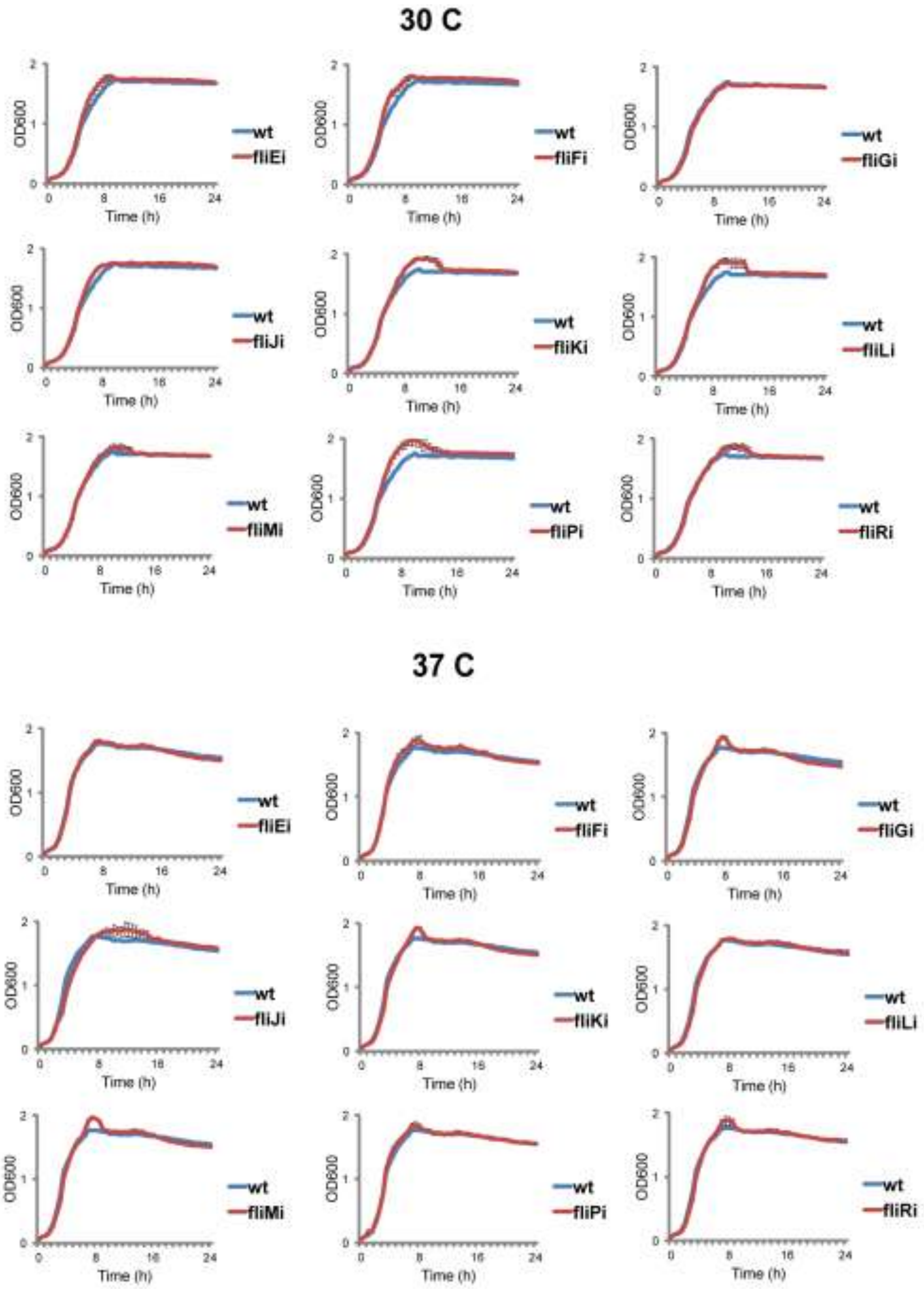
30 C
OD600
Time (h)
wt
fliEi
fliFi
fliGi
fliJi
fliKi
fliLi
fliMi
fliPi
fliRi
37 C
wt
fliEi
fliFi
fliGi
fliJi
fliKi
fliLi
fliMi
fliPi
fliRi

S4 Fig.

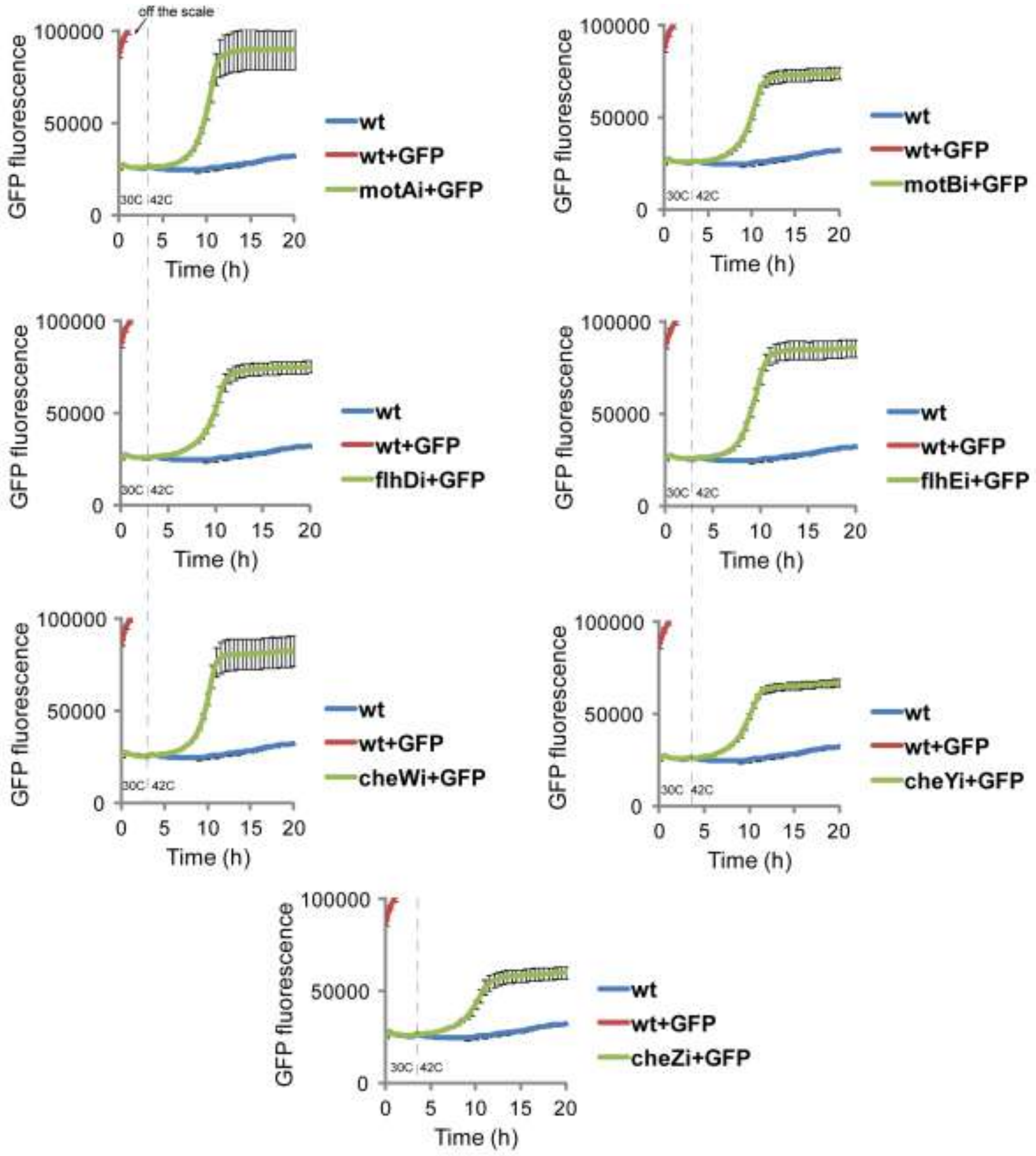
off the scale
GFP fluorescence
100000
50000
0
30C
42C
0
5
10
15
20
Time (h)
wt
wt+GFP
motAi+GFP
motBi+GFP
flhDi+GFP
flhEi+GFP
cheWi+GFP
cheYi+GFP
cheZi+GFP

S5 Fig.

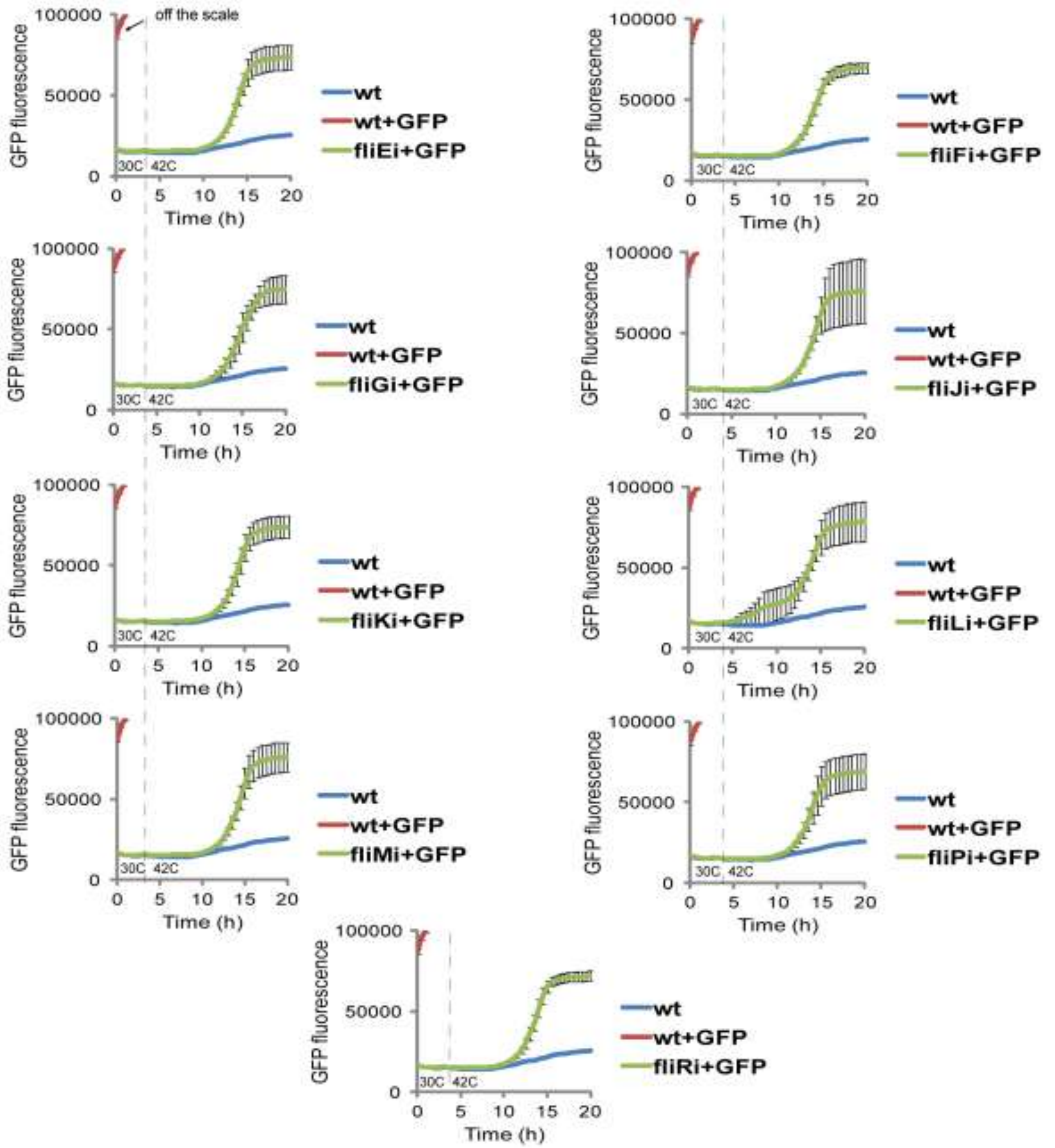
off the scale
GFP fluorescence
Time (h)
30C
42C
wt
wt+GFP
fliEi+GFP
fliFi+GFP
fliGi+GFP
fliJi+GFP
fliKi+GFP
fliLi+GFP
fliMi+GFP
fliPi+GFP
fliRi+GFP

S6 Fig.

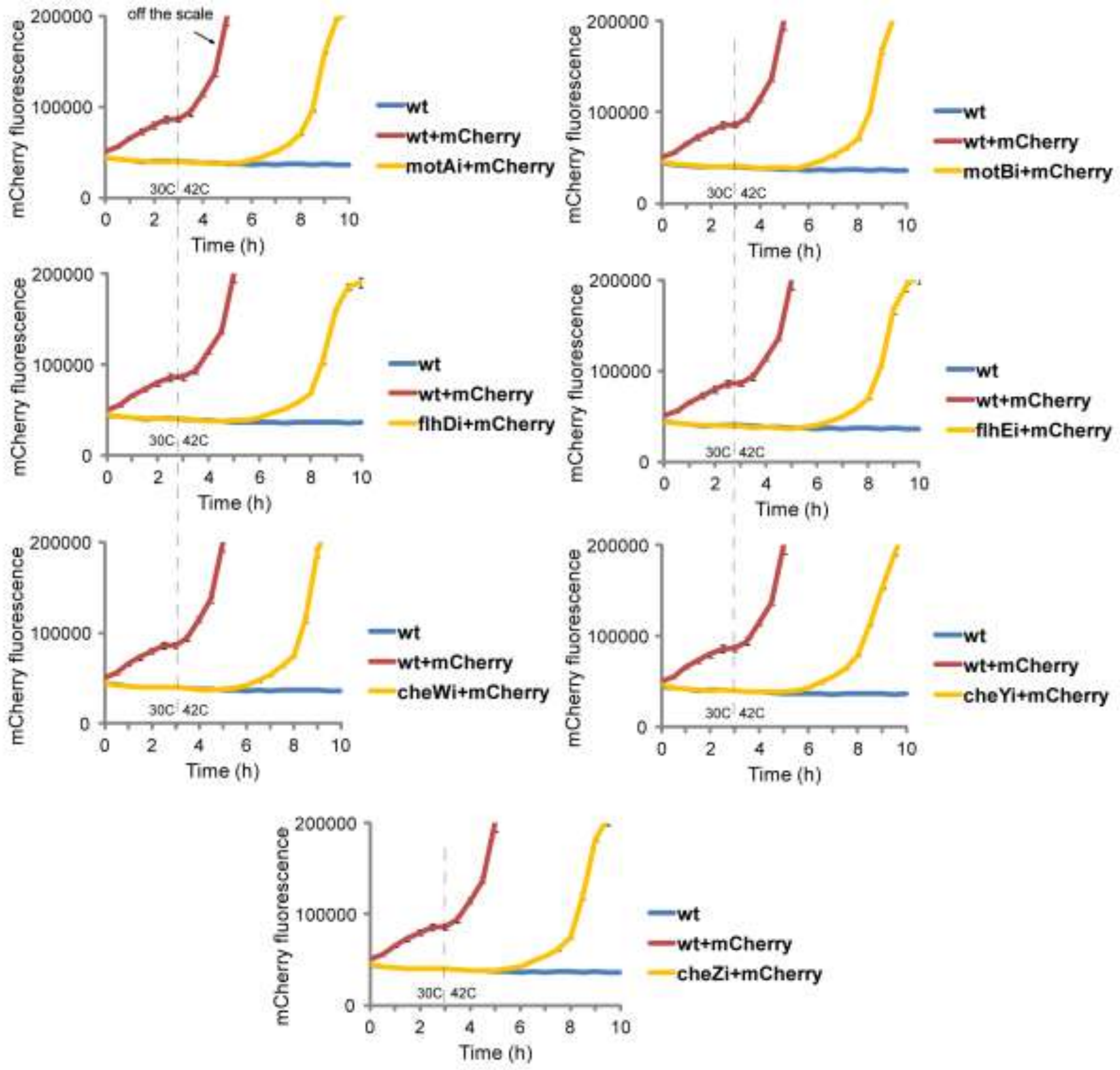
off the scale
mCherry fluorescence
200000
100000
0
0 2 4 6 8 10
Time (h)
30C
42C
wt
wt+mCherry
motAi+mCherry
motBi+mCherry
flhDi+mCherry
flhEi+mCherry
cheWi+mCherry
cheYi+mCherry
cheZi+mCherry

S7 Fig.

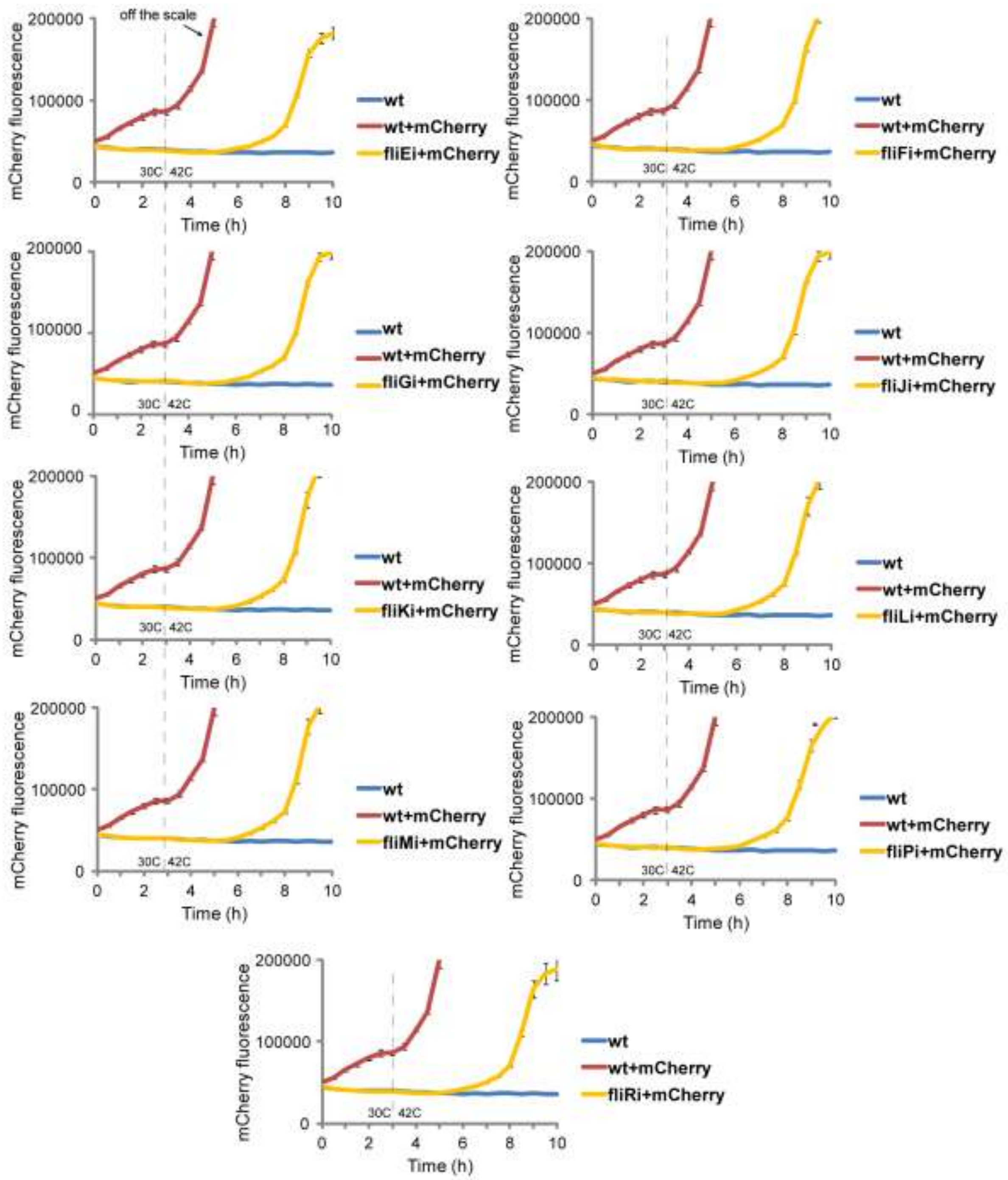
off the scale
mCherry fluorescence
200000
100000
0
30C
42C
0
2
4
6
8
10
Time (h)
wt
wt+mCherry
fliEi+mCherry
fliFi+mCherry
fliGi+mCherry
fliJi+mCherry
fliKi+mCherry
fliLi+mCherry
fliMi+mCherry
fliPi+mCherry
fliRi+mCherry
